# Life stage-specific glycosylation of extracellular vesicles from *Schistosoma mansoni* schistosomula and adult worms drives differential interaction with C-type lectin receptors DC-SIGN and MGL

**DOI:** 10.3389/fmolb.2023.1125438

**Published:** 2023-03-15

**Authors:** Marije E. Kuipers, D. Linh Nguyen, Angela van Diepen, Lynn Mes, Erik Bos, Roman I. Koning, Esther N. M. Nolte-’t Hoen, Hermelijn H. Smits, Cornelis H. Hokke

**Affiliations:** ^1^ Department of Parasitology, Leiden University Medical Center, Leiden, Netherlands; ^2^ Department of Biomolecular Health Sciences, Faculty of Veterinary Medicine, Utrecht University, Utrecht, Netherlands; ^3^ Electron Microscopy Facility, Department of Cell and Chemical Biology, Leiden, Netherlands

**Keywords:** extracellular vesicles, glycans, glycosylation, parasites, schistosoma, C-type lectin receptors, lectins

## Abstract

Schistosomes can survive in mammalian hosts for many years, and this is facilitated by released parasite products that modulate the host’s immune system. Many of these products are glycosylated and interact with host cells *via* C-type lectin receptors (CLRs). We previously reported on specific fucose-containing glycans present on extracellular vesicles (EVs) released by schistosomula, the early juvenile life stage of the schistosome, and the interaction of these EVs with the C-type lectin receptor Dendritic Cell-Specific Intercellular adhesion molecule-3-Grabbing Non-integrin (DC-SIGN or CD209). EVs are membrane vesicles with a size range between 30–1,000 nm that play a role in intercellular and interspecies communication. Here, we studied the glycosylation of EVs released by the adult schistosome worms. Mass spectrometric analysis showed that GalNAcβ1–4GlcNAc (LacDiNAc or LDN) containing N-glycans were the dominant glycan type present on adult worm EVs. Using glycan-specific antibodies, we confirmed that EVs from adult worms were predominantly associated with LDN, while schistosomula EVs displayed a highly fucosylated glycan profile. In contrast to schistosomula EV that bind to DC-SIGN, adult worm EVs are recognized by macrophage galactose-type lectin (MGL or CD301), and not by DC-SIGN, on CLR expressing cell lines. The different glycosylation profiles of adult worm- and schistosomula-derived EVs match with the characteristic glycan profiles of the corresponding life stages and support their distinct roles in schistosome life-stage specific interactions with the host.

## 1 Introduction

Parasitic *Schistosoma* worms infect over 200 million people in Africa alone, and many millions more are infected in tropical areas of the Americas, Eastern Mediterranean, and Western Pacific (Colley et al., 2014). Infection takes place when cercariae, present in infected water, penetrate the skin and develop into egg producing adult worm pairs. Schistosomiasis is treatable with anti-helminthics but reinfection rates are high in endemic areas and there is no vaccine available yet. One of the reasons that *Schistosoma* infections are chronic if untreated and that effective immunity does not develop, is due to the immune modulatory strategies of the parasite that inhibit the generation of adequate protective immune responses (Jenkins et al., 2005). Studying the interaction of this ‘master regulator of the immune system’ with its host can lead to new treatment strategies against schistosome infections. Additionally, the identification of immune regulatory mechanisms utilized by schistosomes may help to identify new treatment opportunities for inflammatory disorders (Maizels et al., 2018).

During each of their life stages schistosomes employ various mechanisms to evade and control host immune responses during an infection. Most of the parasites’ immunomodulatory effects are attributed to excretory/secretory (E/S) products, including glycosylated proteins and lipids. Via specific glycan motifs, these glycoconjugates are known to interact with a range of C-type lectin receptors (CLRs) mainly present on antigen presenting cells of the host. For example, the glycoprotein omega-1 derived from *S. mansoni* eggs enters monocyte-derived dendritic cells (moDCs) *via* binding of Galβ1-4(Fucα1-3)GlcNAc (Lewis X, Le^X^) motifs to the mannose receptor (MR) (Everts et al., 2012). Schistosomula E/S products have also been shown to interact with various other CLRs such as DCIR and DC-SIGN (Bloem et al., 2014), which bind to various fucosylated and mannosylated glycans. The schistosome glycan repertoire also includes GalNAcβ1–4GlcNAc (LacDiNAc or LDN)-motifs, which can be recognized by the macrophage galactose-type lectin (MGL) (van Vliet et al., 2008; Smit et al., 2015b). Since schistosome glycosylation varies between the different life stages and their E/S products, it is likely that differential glycosylation contributes to the differences observed in immune responses induced by schistosomula, adult worms, or eggs.

Recently, we found that the E/S products of *S. mansoni* schistosomula contain extracellular vesicles (EVs) that play a role in pathogen-host interaction *via* uptake by dendritic cells (Kuipers et al., 2020). EVs are lipid membrane vesicles released into the extracellular space by nearly all cells and organisms, including multicellular helminth parasites (Sánchez-López et al., 2021). Generally, EVs are 30–1,000 nm in diameter with an average between 50–150 nm and can contain proteins, lipids, and nucleic acids, which can induce or affect responses by recipient cells. We showed that schistosomula EVs contain both protein- and lipid-linked glycans (Kuipers et al., 2020), corresponding largely to the overall glycan profile of schistosomula (Smit et al., 2015b). Many of these glycans were highly fucosylated and a relatively large proportion carried the Le^X^ trisaccharide, a well-known DC-SIGN ligand. We showed that these glycans mediated schistosomula EV uptake by moDCs mainly *via* interaction with DC-SIGN, leading to augmented cytokine release by the cells. A role for helminth EV-associated glycans in cellular uptake has also been described for *Fasciola hepatica*-derived EVs that displayed a glycosylation-dependent interaction with monocytes (de la Torre-Escudero et al., 2019). Similarly, roles for EV glycans in cancer biology are starting to become apparent (Dusoswa et al., 2019; Nishida-Aoki et al., 2020), including their diagnostic potential (Martins et al., 2021). However, studies on the role of EV glycosylation in parasite-host interaction and detailed studies of EV glycan structures remain limited (Kuipers et al., 2018; Whitehead et al., 2020; Macedo-da-Silva et al., 2021).

It is known that the overall glycan repertoire of *S. mansoni* differs between larvae and adult worms (Smit et al., 2015b). To address whether glycosylation of schistosome-derived EVs is also life-stage dependent, we here elucidated the glycan structures of EVs released by *S. mansoni* adult worms using a mass spectrometry (MS) based approach. The differences in EV-associated glycans between adult worms and schistosomula were confirmed using glycan-targeted monoclonal antibodies (moAbs). Finally, we showed that EV surface glycans significantly influence the interaction of EVs with the MGL or DC-SIGN in CLR expressing cell lines. Our findings suggest that life stage-specific glycosylation of EVs by schistosomal larvae and adult worms influences the interaction of these EVs with host cells and subsequent cellular responses.

## 2 Materials and methods

### 2.1 Parasite culture

**FIGURE 1:**
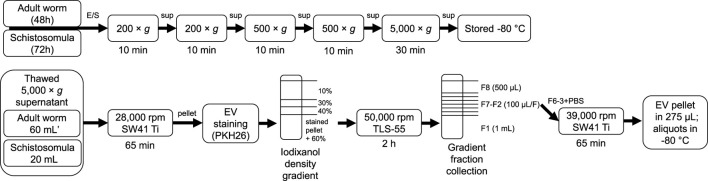
Schematic overview of E/S processing and EV isolation. Summary of the processing of adult worm and schistosomula E/S (top) and subsequent EV isolation (bottom). *additional details on EV isolation for glycan release and western blotting can be found in the corresponding methods sections. E/S, excretory/secretory products; sup, supernatant; min, minutes; h, hours.

Male and female adult worms from the Puerto Rican-strain of *S. mansoni* were obtained through liver perfusion of golden Syrian hamsters (HsdHan-Aura) 7 weeks post infection, in accordance with the Guide for the Care and Use of Laboratory Animals of the Institute for Laboratory Animal Research and have received approval from the university Ethical Review Board (Leiden University Medical Center, Leiden, Netherlands). Collected worms were gently washed at least 5 times with 25–40 mL DMEM (high glucose with L-glutamine, Lonza, Basel, Switzerland) supplemented with Antibiotic Antimycotic Solution (Sigma-Aldrich, St. Louis, MO, United States) and 10 mM HEPES pH 7.4. Dead worms, residual hair or tissue and blot clots were removed. 200–400 worms were cultured in polystyrene culture flasks (75 cm^2^) (Corning, Sigma-Aldrich) in a concentration of 10 worms/mL. After 48 h of culture at 37°C 5% CO_2_, worms were confirmed being viable by microscope and E/S was subsequently collected in 50 mL tubes. The collected E/S was centrifuged twice at 200 × *g* followed by two times at 500 × *g* (all 10 min, 4°C, slow brake, in an SX4750A rotor in an Allegra X-15 R centrifuge) (Beckman Coulter, Brea, CA, United States). The final 500 × *g* supernatant was transferred to 15 mL tubes and centrifuged 30 min at 5,000 × *g* (4°C, max brake) after which the 5,000 × *g* supernatant was transferred to new tubes and stored at −80°C till further use. Cercariae were transformed to schistosomula and cultured as described previously (Kuipers et al., 2020). The E/S from 3 days cultured schistosomula was processed and stored similar as the adult worm E/S using 15 mL tubes in all steps. As a control, supplemented DMEM without parasites was cultured and processed similarly. A summary of E/S processing before EV isolation can be found in Figure 1 (top).

### 2.2 EV isolation and staining

Figure 1 (bottom) shows a schematic overview of the EV isolation steps described in more detail hereafter. The 5,000 × *g* supernatants were thawed overnight at 4°C and transferred to polypropylene tubes (Beckman Coulter). Tubes were centrifuged for 65 min in an SW41 Ti rotor at 28,000 rpm (average 96,808 × *g*, k-factor 265) at 4°C in an XE90 centrifuge (Beckman Coulter). For one adult worm EV or schistosomula EV isolation, 60 mL (equal to material from 600 worms) or 20 mL (equal to material from 150,000 schistosomula) of 5,000 × *g* supernatant was used, respectively. The EV-enriched pellets were resuspended and pooled in 20–60 µL PBS/0.2% BSA (made from 5% BSA in PBS, top 2/3 supernatant from >16 h centrifuged at 28,000 rpm (96,589 × *g*, k-factor 266) in an SW32 rotor) and transferred to a TLS-55 polypropylene tube. Diluent C was added to the resuspended EV pellet to obtain a total volume of 100 μL, to which 93 μL diluted PKH26 (Sigma-Aldrich) (1.5 μL in 100 μL Diluent C) was added. After 3 min, the staining reaction was quenched by the addition of 100 µL RPMI/10% EV depleted FCS (top 2/3 supernatant from RPMI/30% FCS centrifuged >16 h at 28,000 rpm in SW32 rotor). Stained pellets (293 μL) were gently mixed with 660 μL 60% iodixanol (Optiprep, Axis-Shield PoC AS, Oslo, Norway) and a gradient was built on top with 220 μL 40%, 220 μL 30%, and 720 μL 10% iodixanol. Gradients were centrifuged in an Optima TLX centrifuge (Beckman Coulter) for 2 h at 50,000 rpm (average 166,180 × *g*, k-factor 60) (slow acceleration and deceleration) and 4°C in a TLS-55 rotor. Fractions were collected from the gradient from top to bottom by pipetting one time 500 µL (F8) followed by six times 100 µL (F7-2) and a remaining 1 mL bottom fraction (F1). The refractive index (RI) of the fractions were measured with a CETI refractometer (Medline Scientific, Chalgrove, UK) (15 µL per fraction) and densities were calculated with the formula [3,35*(RI)]-3,4665. F6-3 were pooled in an SW41 tube and topped up with cold PBS. The tube was covered with parafilm and decanted for 10 times after which the tube was spun at 39,000 rpm (average 187,813 × *g*, k-factor 136) in an SW41 Ti rotor for 65 min (4°C). The purified EV pellet was resuspended in 275 µL PBS and aliquots were stored at −80°C. Medium control was processed similarly as control for unbound dye.

Protein concentration of a thawed aliquot of purified EV was measured according to the manufacturers protocol for microBCA (Pierce, Thermo Fisher Scientific, Waltham, MA, United States). For nanoparticle tracking analysis (NTA), aliquots were thawed from −80°C and 100 times diluted in PBS directly before NTA measurement. Each sample was recorded three times 30 s on camera level 12, 14, and 16 on a NanoSight NS500 (Malvern Panalytical, Malvern, UK) equipped with an sCMOS camera. The analysis and average particle concentration calculation was performed as previously described (Kuipers et al., 2020). All relevant data of our experiments have been submitted to the EV-TRACK knowledgebase (EV-TRACK ID: EV220409) (Van Deun et al., 2017).

### 2.3 Cryo electron microscopy

EVs were purified on a density gradient as described above. The EV containing fractions with a density between 1.21–1.07 g/mL were pooled (total volume of 580 µL) and washed with PBS on a 0.5 mL 10 kDa centrifugal filter (Amicon, Merck KGaA, Darmstadt, Germany) as described (Kuipers et al., 2022). The sample (3 µL) was subsequently applied on a 300 mesh EM grid (Quantifoil R2/2, Jena, Germany) that was previously glow-discharged (2 min in 0.2 mbar air using a EMITECH K950X with glow discharger unit) and vitrified using an EMGP (Leica, Wetzlar, Germany) at room temperature and 100% humidity. Excess sample was removed by blotting once for 1 s with filter paper (Whatman #1). The blotted grid was plunged into liquid ethane (−183°C). After vitrification, the grid was stored under liquid nitrogen until further use. The grid was mounted in a Gatan 626 cryo-holder for cryo-EM imaging. Cryo-EM imaging was performed on a Tecnai 12 electron microscope (FEI Company, Eindhoven, Netherlands) operated at 120 kV. Images were recorded on a 4k×4k Eagle camera (FEI Company) at ×18,000 magnification (pixel size 1.2 nm) between 5 and 10 µm under focus.

### 2.4 Glycan release

Iodixanol purified EVs from 600 adult worms were used for the release of total N-glycans as previously described (Smit et al., 2015b). Briefly, lyophilized EVs were sonicated and denatured before 24 h N-glycosidase (PNGase) F (4 U/100 μL, Roche Diagnostics, Almere, Netherlands) treatment. Released N-glycans were isolated and purified using reversed phase (RP) C18-cartridges (JT Baker, Phillipsburg, NJ, United States) loaded with the PNGase F treated sample followed by loading the flow through on carbon cartridges (Supelclean ENVI-carb SPE, Sigma-Aldrich) as described (Petralia et al., 2022). Additionally, intact purified EVs without prior denaturation were treated with PNGase F for 24 h at 37°C, after which the EVs were resuspended with PBS in a TLS-55 tube and spun for 65 min (4°C) at 42,000 rpm (average 117,553 × *g*, k-factor 87) in an Optima TLX centrifuge. N-glycans released in this way from the EV surface were isolated from the supernatant and the EV pellet was resuspended and treated as above to obtain the remaining EV associated glycans that were not released by PNGase F. To obtain glycolipid derived glycans, purified as well as enriched adult worm EVs were treated with endo-glycoceramidase as described previously (Kuipers et al., 2020). All isolated glycans were labelled with 2-aminobenzoic acid as described (Petralia et al., 2022).

### 2.5 Glycan analysis

Isolated glycans were analyzed using Matrix Assisted Laser Desorption/Ionisation–Time of flight mass spectrometry (MALDI-TOF-MS) using a Bruker Daltonics UltrafleXtreme^®^ mass spectrometer equipped with a 1 kHz Smartbeam II laser and controlled by the software FlexControl 3.4 Build 119, as previously reported (Petralia et al., 2022). 2-AA labelled glycans solubilized in MQ were mixed onto a 384-well polished steel target plate with 2,5-dihydroxybenzoic acid (DHB) matrix (#8201346, Bruker Daltonics, 20 mg/mL in 30% ACN) while products of exoglycosidase digestions were directly eluted onto the plate in 50% ACN, 0.1% TFA mixed with DHB (10 mg/mL) at the end of the enzyme removal with C18 Millipore^®^ Zip-Tips. All spectra were obtained in the negative-ion reflectron mode using Bruker^®^ peptide calibration mix (#8206195, Bruker Daltonics) for external calibration. Spectra were obtained over a mass window of *m/z* 700–3,500 with ion suppression below *m/z* 700 for a minimum of 20,000 shots (2000 Hz) obtained by manual selection of “sweet spots”. The software FlexAnalysis (Version 3.4 Build 50) was used for data processing including smoothing of the spectra (Savitzky Golay algorithm, peak width: *m/z* 0.06, one cycle), baseline subtraction (Tophat algorithm) and manual peak picking. Peaks with a signal-to-noise ratio below three were excluded as well as known non-glycan peaks or contaminating glucose polymers. Deprotonated masses of the selected peaks were assigned using the GlycoPeakfinder^®^ tool of the free software GlycoWorkBench (Version 3, 29 June 2007) (Ceroni et al., 2008). The 2-AA label was taken into account as a fixed reducing-end modification and possible glycan composition was set up based on available schistosome glycosylation data in the literature (Smit et al., 2015b). A deviation of 300 ppm was allowed for initial assignment of compositions. Spectral assignments were aided by additional MS/MS measurements, exoglycosidase sequencing, and available published structural data (Smit et al., 2015b). MS/MS was performed for structural elucidation *via* fragmentation ion analysis by MALDI-TOF/TOF on selected ions using the UltrafleXtreme^®^ mass spectrometer in negative-ion mode. Confirmation of structures was also aided by treatments of N-glycans with β-N-acetylglucosaminidase from *Streptococcus pneumoniae* and β-N-acetylhexosominidase from *Streptomyces plicatus* (New England Biolabs, Ipswich, MA, United States; P744, P721, respectively). Enzymatic digestions were performed by digesting 1–2 µL of 2-AA labelled glycan overnight at 37°C in recommended buffer in 10 µL total reaction volumes.

### 2.6 Western and lectin blotting

EV-enriched pellets from adult worm E/S (per gradient from 300–600 worms) or schistosomula E/S (per gradient from 110,000–220,000 schistosomula) were resuspended in 73 µL PBS/0.2% BSA, transferred to TLS-55 tubes, and gently mixed with 440 µL 60% iodixanol. The gradient was built by carefully loading 220 μL 40%, 220 μL 30%, and 792 μL 10% iodixanol on top and spun as described above. After centrifugation, 12 equal fractions of 145 µL were collected from top to bottom, 15 µL was used for determining the refractive index, and 125 µL was directly mixed with 42 µL 4× non-reducing sample buffer (0.2 M TrisHCl pH 6.8, 8% SDS, 40% glycerol, and Bromophenol blue). In addition, the 96,589 × *g* EV-depleted supernatant of the E/S was concentrated in 10 kDa filter tubes (Amicon) to 300.9 µL to which 100.2 µL 4× non-reducing sample buffer was added. This mix was 5 times diluted in sample buffer to load an equal end volume compared to all the fractions of one gradient. All samples were heated at 98°C for 3 min and stored at −20 °C till SDS-PAGE.

15 µL of sample or 1.5 µL of marker (PageRuler Plus, Thermo Fisher Scientific) was run into a 12.5% gel, blotted onto PVDF membranes and blocked with blocking buffer consisting of PBS supplemented with 0.1% Tween-20% and 0.2% gelatin from cold water fish skin (Sigma-Aldrich). Blots were incubated overnight with antibodies (1:200–1:2000) in blocking buffer. Antibodies used included: TSP2-2D6 (kind gift from prof. Alex Loukas, James Cook University, Australia) and monoclonal antibodies generated in house at LUMC (Smit et al., 2015a): 100-4G11 (van Remoortere et al., 2003), 114-4D12, 128-1E7, 258-3E3, 273-3F2, 290-2E6, and 291-5D5. Bands were visualized with chemiluminescence substrate (SuperSignal West Pico PLUS, Thermo Fisher Scientific) in an Alliance Q9 (UVITEC, Cambridge, UK) and analysed with Fiji/ImageJ (Schindelin et al., 2012).

For the lectin blots, 260 µL of EV enriched pellets from 1,200 adult worms was split equally (130 µL) and incubated with or without PNGase F (4 U/100 µL) for 24 h at 37°C. EVs were subsequently purified with an iodixanol gradient and fractions 4–7 (1.08–1.20 g/mL) were pooled and washed as described above. Final pellets were resuspended in 70 µL PBS and mixed with 2× non-reducing sample buffer, heated, and stored as described. Similar SDS-PAGE and blotting was performed as above. Blocking buffer for the lectin blots consisted of TBS supplemented with 5% BSA and 0.1% Tween-20. Blots were blocked overnight and subsequently incubated for 1 h with 5 μg/mL biotinylated SBA (*Glycine* max (soybean) agglutinin) or DBA (*Dolichos biflorus* agglutinin) (Vector laboratories, Burlingame, CA, United States). Visualization was done as described above after 30 min of Streptavidin poly-HRP (Sanquin, Amsterdam, Netherlands) incubation and several washing steps.

### 2.7 PNGase F treatment of EVs for cell experiments

EV-enriched pellets, obtained as described above from 120 mL adult worm (material from 1,200 worms) or 40 mL schistosomula (from 300,000 schistosomula) 5,000 × *g* supernatant, were resuspended in a total volume of 150 µL or 100 µL PBS with 0.2% BSA (as above), respectively. Pellets were then split in two separate Eppendorf tubes and 4 µL PNGase F (4 U) was added to one of these two tubes. All samples were incubated for 20 h at 37°C after which the EV were stained with PHK26 and isolated *via* a iodixanol density gradient as described above. The final purified EV pellets were resuspended in 175 µL PBS and stored at −80°C till further use.

### 2.8 C-type lectin receptor cell lines

CHO and CHO-MGL (kind gift from dr. ing. S. J. van Vliet (van Vliet et al., 2005) cells were maintained in RPMI supplemented with 10% FCS, L-glutamine (20 µM), penicillin (100 U/mL), and streptomycin (100 U/mL). K562 and K562-DC-SIGN cells (kind gift from prof. dr. C.G. Figdor (Geijtenbeek et al., 2000) were maintained in DMEM with 10% FCS, L-glutamine, pyruvate (20 µM), penicillin, and streptomycin. Geneticin (0.6 mg/mL) (G418, Roche Diagnostics) was added to the CHO-MGL and K562-DC-SIGN cultures to select for cells with the CLR expressing vector. Cells (400,000/mL) rested for 2 h (37°C, 5% CO_2_) after harvest before addition of PKH26 labelled EVs or dye control with or without 30 min EGTA (10 mM) pre-incubation. After 18 h incubation, cells were placed on ice, harvested, stained with Aqua live/dead staining (Invitrogen, Thermo Fisher Scientific), and measured on a FACSCanto II (BD Bioscience, Franklin Lakes, NJ, United States). Receptor expression was measured by staining cells with αCD209-V450 (clone DCN46, BD Biosciences), αCD301-APC (clone H037G3, Biolegend, San Diego, CA, United States), αFcgammaR-binding inhibitor (eBioscience, Invitrogen, Thermo Fisher Scientific), and 7AAD viability dye (eBioscience).

Data was statistically analyzed in GraphPad Prism 8.0 (GraphPad Software Inc., La Jolla, CA, United States) using a repeated measure One-way ANOVA with Dunnett’s Multiple Comparison Test. *p* values < 0.05 were considered significant.

## 3 Results

### 3.1 Characterization of adult worm EVs by cryo-EM and NTA

**FIGURE 2:**
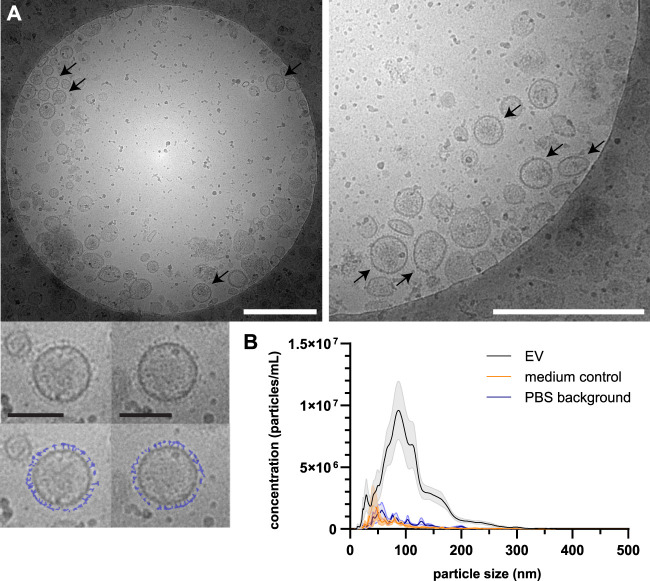
Isolated EVs from Schistosoma adult worms visualized by cryo-EM and measured by NTA. Purified EVs were visualized by cryo-EM **(A)** showing surface structures (corona) on part of the EVs (arrows). The bottom panel shows two individual EVs with a copy in which the surface structure is drawn in blue. White scale bars are 500 nm, black scale bars are 100 nm. NTA was performed on purified adult worm EVs from eight biological replicates of which the average is shown (black line) with SEM (grey area) **(B)**. In addition, four medium controls (orange) and four samples PBS (background) in which the EVs were resuspended (blue) are shown.

*Schistosoma mansoni* adult worm EVs were isolated from E/S collected following a 48 h culture of adult worms using sequential (ultra)centrifugation and density gradient purification to separate EVs from non-EV particles. EV preparations were visualized by cryo-EM and showed the presence of 30–180 nm vesicles (Figure 2A). Part of these EVs had small structures extruding from their surface (Figure 2A, arrows and bottom panel). NTA of the adult worm EVs showed a size-range between 30 and 250 nm with the majority of the EVs to be around 90 nm in size (Figure 2B). The small peaks of particles observed in the medium control (similarly processed culture medium without parasites) and in PBS alone, were considered background noise of the NTA. The average number of particles measured was equal to 6.85 E^9^ per 100 adult worms with an average of 4.89 µg protein from EVs of 100 adult worms.

### 3.2 EV surface contains glycan motifs prevalent in adult worms

**FIGURE 3:**
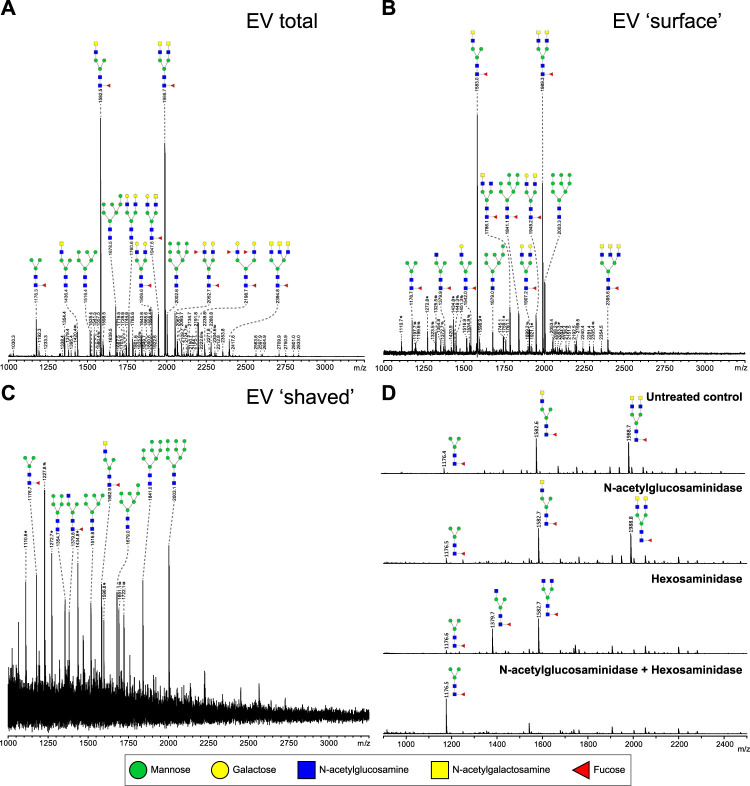
Most relative abundant complex N-glycans on adult worm EVs contain LDN-motif. N-glycans released by PNGase F of total **(A)**, intact (‘surface’ N-glycans accessible to the enzyme) **(B)**, and PNGase F treated (‘shaved’, N-glycans on surface or inside EV that PNGase F cannot access) **(C)** adult worm EVs were measured by MALDI-TOF-MS. To confirm the presence of GalNAcβ1-4GlcNAc (LDN) antennae in glycans released from EV surface in B, AA-labeled N-glycans were treated with either β-N-acetylglucosaminidase, β-hexosaminidase, or both. β-N-acetylglucosaminidase removes GlcNAc residues only after they become accessible by β-hexosaminidase digestion to remove the terminal β-linked GalNAc residues. **(D)** Peaks are labelled with their monoisotopic masses. The structural assignments are putative and were deduced from the masses (which includes the 2-AA label) and previously published data (Smit et al., 2015b). These are representative spectra for three biological replicates. Green circle, mannose; yellow circle, galactose; blue square, N-acetylglucosamine; yellow square, N-acetylgalactosamine; red triangle, fucose. *, signals corresponding to hexose oligomer of unknown origin; #, non-glycan signals.

To study glycosylation of adult worm EVs, the isolated N-glycans and glycolipid-derived glycans were analyzed by mass spectrometry. Glycan structures were assigned to each of the observed molecular ions using the previously published whole adult worm glycan profiles as reference library (Smit et al., 2015b). The N-glycan composition of the adult worm EVs (Figure 3A) revealed a similar complex glycan profile as described for 6-week old adult worms (Smit et al., 2015b). The two major ion peaks in the spectrum represented core (α6)-fucosylated di-antennary structures containing the LDN motif on either one or both antennae. Less abundant N-glycans observed in the spectrum were oligomannosidic structures (3–9 mannoses), glycans with one or two antennae consisting of Galβ1–4GlcNAc (LacNAc, LN), or an LN and LDN combination, and two minor peaks with structures containing an LN and Le^X^ or two Le^X^ motifs. Each of these N-glycans were previously confirmed to be present in whole adult worm extracts (Smit et al., 2015b). In all repeated analyses, glycolipids were not detectable in the adult worm EVs (data not shown).

Glycans present on the EV surface can form ligands for host lectins that play a role in the activation and modulation of immune responses (van Die et al., 2003; van Remoortere et al., 2003; Everts et al., 2012). To identify these surface glycans, intact purified adult worm EVs were first treated with PNGase F to release and collect EV surface N-glycans. The remaining ‘shaved’ EVs were re-pelleted, disrupted by sonication and denaturing, and then again treated with PNGase F to release the N-glycans present on the inside of the shaved EVs. The most abundant N-glycans at the EV surface (Figure 3B) were the same LDN-containing structures that were most abundant in the total EV N-glycan profile. The minor peaks observed also represented similar structures with LN or LN-LDN antennae as observed in the total spectrum. In the shaved EV however, oligomannosidic structures (3–9 mannoses) were the most abundant, and complex-type N-glycans were not detected (Figure 3C). The occurrence of a single LDN antenna rather than two separate GlcNAc terminating branches in the N-glycan species observed at *m/z* 1,582.6, and occurrence of two LDN antenna in the *m/z* 1988.7 structure, were confirmed by treatments with *N-*acetylglucosaminidase and *N-*acetylhexosaminidase (Figure 3D) and by MS/MS fragmentation analysis (Supplementary Figure S1). These results indicate that *S. mansoni* adult worm EVs expose N-glycans with one or two terminal LDN motifs.

### 3.3 Difference in EV-associated glycoconjugates between adult worms and schistosomula

**FIGURE 4:**
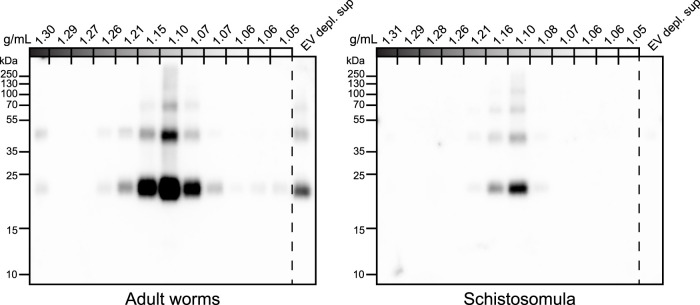
Tetraspanin-2 containing EV fractions in iodixanol gradient. EV enriched pellets were floated upwards into iodixanol density gradients. Twelve equal fractions were obtained, mixed with sample buffer and loaded on an SDS-PAGE gel followed by western blot targeting *Schistosoma* Tetraspanin-2 (TSP2). EV depl. sup = EV depleted supernatant (100,000 × *g* supernatant that was concentrated to equal volume of the full density gradient).

To confirm the glycan data obtained by mass spectrometry [Figure 3 and (Kuipers et al., 2020)], and make a direct comparison with schistosomula EVs, we investigated glycosylation of both adult worm and schistosomula EVs by western blotting. Monoclonal antibodies (mAbs) directed against various glycan motifs including LDN, the unsubstituted tri-mannosyl core motif, and fucosylated (F)-GlcNAc (Supplementary Figure S2) were used to visualize glycoproteins with these motifs in their N-glycans, as well as in O-glycans possibly present but undetected by our MS approach. An overview of the detected glycans in EVs and EV-depleted supernatant of both life stages (as analyzed in Supplementary Figure S2A) is provided in Table 1. The presence of the EV marker Tetraspanin-2 (TSP2) confirmed that the majority of the EVs from adult worms and schistosomula were present in fractions with a density of iodixanol between 1.07 and 1.16 g/mL (Figure 4). For the adult worms, minute amounts of TSP2 were also detected in the bottom fraction and top fractions and in the EV-depleted supernatant.

**TABLE 1:** Glycan motifs in EV or EV-depleted supernatant from adult worms and schistosomula detected with western Blot. –: not detected; +: 1–50,000; ++: 50,000–200,000; +++: >200,000 area under the curve measured in ImageJ. Data based on blots in Supplementary Figure S2B. LDN, GalNAcβ1-4GlcNAcβ1-; LDN-F, GalNAcβ1-4(Fucα1-3)GlcNAcβ1-; F-GlcNAc, Fucα1-3GlcNAcβ1-; F-LDN, Fucα1-3GalNAcβ1-4GlcNAcβ1-; F-LDN-F, Fucα1–3GalNAcβ1–4(Fucα1–3)GlcNAcβ1-; DF-GlcNAc, Fucα1-2Fucα1-3GlcNAcβ1-; TF-GlcNAc, Fucα1-2Fucα1-2Fucα1-3GlcNAcβ1-.

	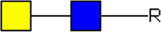 LDN	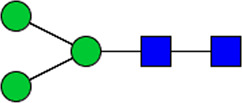 tri-mannosyl core	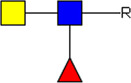 LDN-F	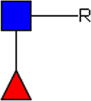 F-GlcNAc	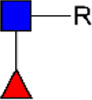 F-GlcNAc	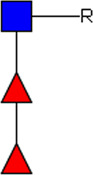 DF-GlcNAc	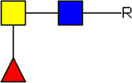 F-LDN
				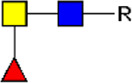 F-LDN		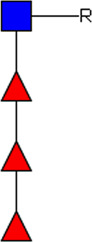 TF-GlcNAc	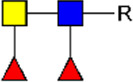 F-LDN-F
				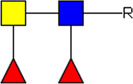 F-LDN-F			
Adult worm EV	+	+	++	-	-	-	-
Adult worm EV-depleted supernatant	+	+	+	-	-	-	-
Schistosomula EV	-	-	+	++	++	+++	++
Schistosomula EV-depleted supernatant	-	++	+	+	+	+	+++

The most abundant glycan motifs detected in the adult worm EVs were LDN, the tri-mannosyl motif, and LDN with a GlcNAc-linked fucose [GalNAcβ1-4(Fucα1-3) GlcNAc, LDN-F] (Table 1). These same motifs were also detected in the EV-depleted supernatant, however, with a different band pattern (Supplementary Figure S2B), suggesting that the EVs and EV-depleted supernatant contain different glycoprotein subsets. MAbs targeting fucosylated GalNAc, such as Fucα1–3GalNAcβ1–4(Fucα1–3)GlcNAc (F-LDN-F), or fucosylated GlcNAc (Fucα1-3GlcNAc, F-GlcNAc) were not reactive in western blots of adult worm EV samples. The diverse glycosylation of schistosomula EVs, previously characterized by mass spectrometry (Kuipers et al., 2020), was illustrated using western blotting by the substantial recognition of F-LDN, LDN-F, and F-GlcNAc motifs (Table 1). In accordance with the previous findings, unsubstituted LDN was not detected in schistosomula EV. Interestingly, the tri-mannosyl was abundantly detected in the schistosomula EV-depleted supernatant (Supplementary Figure S2B). Other glycan specific band patterns in the EV-depleted supernatant were also distinct from the patterns observed in the EV fractions. Together, these findings suggest that EVs contain glycoproteins distinct from those in non-EV E/S products. Moreover, the western blot data confirm along with the mass spectrometry data that schistosomula and adult worm EVs are differentially glycosylated, in line with the overall differential glycosylation of these schistosome life stages (Smit et al., 2015b).

### 3.4 Adult worm EVs interact with MGL receptor

**FIGURE 5:**
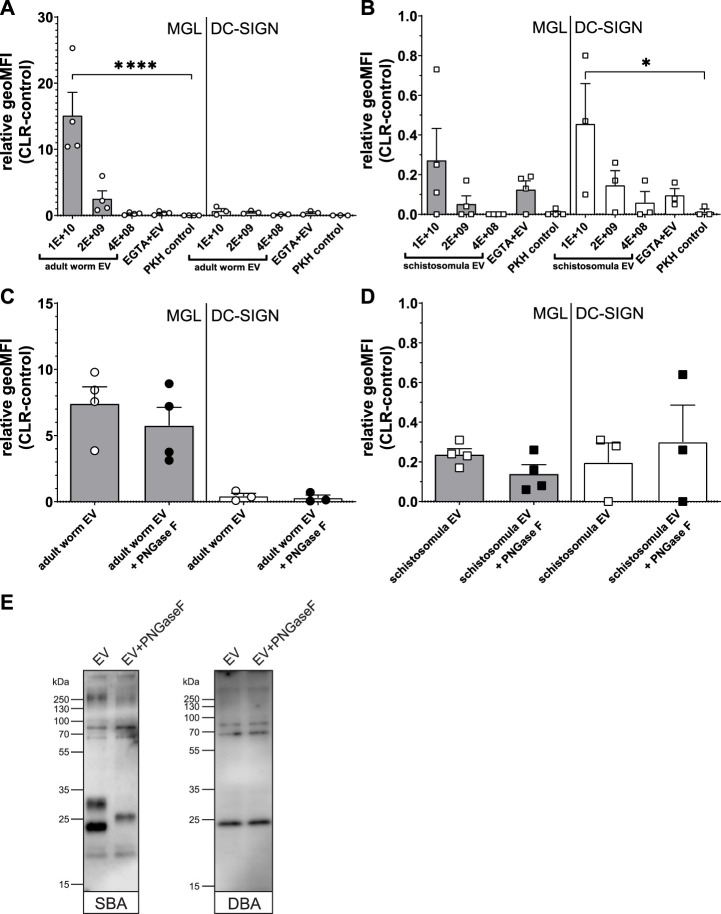
Interaction *Schistosoma* EVs with MGL or DC-SIGN expressing cell lines suggest multiple MGL ligands on adult worm EVs. Fluorescently labelled EVs from adult worms **(A)** or schistosomula **(B)** or dye (PKH) control were incubated with MGL or DC-SIGN expressing cell lines and measured for fluorescence by flow cytometry. The geometric mean fluorescent intensity (geoMFI) of similar cell lines without CLR expressing vector incubated with EVs (control) was subtracted from the geoMFI of the cells with the CLR. Similar experiment was performed with EVs treated with PNGase F before density gradient isolation **(C, D)**. Adult worm EVs treated with or without PNGase F were loaded on SDS-PAGE followed by western blot for detection of LDN (SBA lectin) and Tn antigen (DBA lectin) **(E)**. EV concentrations are given in EV/mL. Data from three to four independent experiments. Mean ± SEM *p < 0.05, ****p < 0.0001, using repeated measures ANOVA with Dunnett’s Multiple Comparison Test compared to PKH control.

Previously, we found that *S. mansoni* schistosomula EVs interact with DC-SIGN, but not with MR or DCIR, which was in line with the clear presence of DC-SIGN ligands on the schistosomula EV surface (Kuipers et al., 2020). Since the most abundant N-glycans on the surface of the adult worm EVs contained LDN (Figure 3C), we reasoned that adult worm EV could bind to MGL rather than DC-SIGN. MGL is a CLR expressed on monocyte-derived DCs and macrophages, capable of binding LDN- and GalNAc-containing glycoconjugates present in helminths and tumors (van Kooyk et al., 2015). Indeed, flow cytometric measurements of cell lines overexpressing the MGL or DC-SIGN receptor (Supplementary Figure S3) showed a significant interaction of MGL with fluorescently labelled adult worm EVs, while these EVs did not interact with DC-SIGN expressing cells (Figure 5A). The opposite was observed for the schistosomula EVs, which were strongly interacting with DC-SIGN but less with MGL (Figure 5B). These EV-CLR interactions were confirmed to be glycan-dependent, as the pre-incubation of EGTA disabling the Ca^2+^ dependent glycan binding domain of CLR, reduced the binding or uptake of EVs.

Since we observed that the LDN-containing N-glycans on the surface of the adult worm EVs could be removed by PNGase F (Figure 3D), we studied the effect of PNGase F treatment of EVs on MGL-dependent binding. Surprisingly, PNGase F treatment had no significant effect on adult worm EVs binding or uptake by the CLR expressing cells (Figure 5C). Similarly, PNGase F treatment did not affect schistosomula EVs binding to DC-SIGN (Figure 5D), which we attributed earlier to the presence of additional and abundant DC-SIGN ligands within the schistosomula EV-associated glycolipids (Kuipers et al., 2020). We did not detect any glycolipids in the adult worm EVs, however, to possibly explain the MGL affinity remaining after removal of LDN-containing N-glycans. Therefore, we investigated by lectin blotting whether PNGase F treatment resulted in complete LDN removal as expected (Figure 3C) and whether the adult worm EVs might also contain other MGL ligands such as Tn antigens. The Tn antigen is an O-linked α-GalNAc residue often occurring in mucin domains and a known MGL ligand produced by various organisms, including mammals and helminths (van Vliet et al., 2005). Lectin blotting with SBA, which recognizes both α-GalNAc (as in Tn) and β-GalNAc (as in LDN), showed that PNGase F treatment did indeed affect N-glycan contained LDN reactivity. The band around 23 kDa was absent in the PNGase F treated EV lane, and the band at ∼30 kDa disappeared while a new band showed at ∼26 kDa (Figure 5E), likely due to removal of N-glycans resulting in a mass shift. The remaining SBA binding after PNGase F treatment suggests that other SBA ligands than N-glycan LDN, such as O-linked glycans with the LDN motive or the Tn antigen are present. Application of DBA, which binds only α-GalNAc, confirmed that glycoproteins of adult worm EVs indeed contain Tn antigen, and that these are not affected by PNGase F treatment. These findings suggest that the interaction of PNGase F treated, N-glycan shaved, adult worm EVs with MGL are most likely due to the presence of O-linked GalNAc residues exposed on the EVs.

## 4 Discussion

Helminth parasites, including *S. mansoni*, release EVs that can interact with host cells. There are multiple publications reporting about the proteome and transcriptome of adult schistosome (Sotillo et al., 2016; Meningher et al., 2017; Samoil et al., 2018; Kifle et al., 2020). However, the glycans associated with these worm EVs remain largely undescribed and their role has been almost completely unexplored so far (Kuipers et al., 2020; Dagenais et al., 2021). Here we found that EV released by adult worms contained complex N-glycans with LDN motifs, which were almost exclusively present on the EV surface. The observed EV-glycan profile corresponds with the glycan profile of entire adult worms (Smit et al., 2015b). This similarity between the overall glycan profile of a life stage and the glycan profile of their released EVs has been previously observed for schistosomula as well (Kuipers et al., 2020). This indicates that the previously observed differences in overall N-glycan profile between the two life stages (Smit et al., 2015b) are reflected in their released EVs [Figure 3A and (Kuipers et al., 2020)], where adult worm EV N-glycans mainly contain LDN, but not highly fucosylated glycans as observed for schistosomula EVs. This was confirmed by both mass spectrometry (Figure 3) and glycan-targeting antibodies in a western blot analysis (Table 1, Supplementary Figure S2). In contrast to a recent publication on *S. mansoni* adult worm EVs that were analyzed by lectin microarrays (Dagenais et al., 2021), we did not detect sialylated host-derived glycans in our EV preparations (Figure 3). We did not use FCS in worm culture medium and applied multiple washing steps before EV analysis to exclude the presence of host material. Helminths, such as *S. mansoni*, lack the molecular machinery for the biosynthesis of sialylated glycans (Hokke and van Diepen, 2017). Although our data shows that schistosomes in an *ex vivo* culture do not release EVs with sialylated glycan motifs, it could be possible that host glycoproteins are incorporated into schistosome EVs *in vivo,* or that host-derived sialic acids get incorporated into schistosome products otherwise (Dagenais et al., 2022).

The majority of the EV-associated glycoprotein glycans we detected have previously been reported to be present in whole adult worm extracts. We could, however, not detect glycolipids known to be abundantly present in adult worms (Smit et al., 2015b) in the purified worm EVs. In contrast, glycolipids are ample and diverse in the case of schistosomula-derived EVs (Kuipers et al., 2020). Furthermore, the unique long filamentous ‘hair-like’ structures previously observed on the surface of schistosomula EVs (Kuipers et al., 2020) were not present on adult worm EVs (Figure 2A). These observations could suggest a difference between schistosomula and adult worms in cellular source and pathways from which the EVs derive. There is evidence that schistosomula EVs are released from their pre-acetabular glands (Gasan et al., 2021), although they may also origin from the cercarial glycocalyx, as their long surface filaments resemble the glycocalyx morphology (Samuelson and Caulfield, 1985). The source of adult worm EVs is largely unknown but we suggest that the adult worm gut and tegument are likely candidates as a high abundance of LDN structures has been reported for both locations using lectin staining (Schmidt, 1995). In addition, the EV marker TSP2 has been found abundantly on the adult worm tegument (Tran et al., 2006). Both tegument and gut-derived EVs have been observed or suggested to occur for other helminths (Drurey et al., 2020), but the exact adult *S. mansoni* worm EV source or sources remain(s) to be elucidated. The corona structure on adult worm EVs (Figure 2A) appears to be a unique feature. The EV (protein) corona gained more interest just recently (Buzas, 2022) and to our current knowledge the structures observed on the adult worm EV surface have not been imaged/reported as such on the surface of naturally released mammalian EVs in the absence of blood plasma. Since we did not detect host-derived sialic acids in our measurements, it is also unlikely that the worm EV corona observed consists of host material, yet the exact composition remains to be elucidated. These coronas do resemble the surface structures seen on EVs from transfected mammalian cells enriched in nematode or viral membrane proteins (Zeev-Ben-Mordehai et al., 2014). Furthermore, coated EVs have been reported for other helminths (Sánchez-López et al., 2020), fungi (Rizzo et al., 2021), and bacteria (Gui et al., 2016; Cecil et al., 2019). It is therefore tempting to suggest that these membrane structures are a hallmark of pathogen-derived EVs and may be involved in pathogen-host interaction.

The finding that adult worm EVs interacted more significantly with MGL compared to DC-SIGN, while for the schistosomula EV the opposite was found (Figure 5) indicates that the glycosylation of *S. mansoni* EVs drives their interaction with host cells. Both receptors are selectively present on DCs and macrophages but rarely coincide on the same cell *in vivo* and have only been found together on some cells in the small intestine (van Vliet et al., 2008), suggesting distinct immunological functions. DC-SIGN can bind glycoconjugates containing either mannose or fucose structures which, in specific molecular context and with additional TLR activation, can promote Th1 and Th2 responses, respectively (Geijtenbeek and Gringhuis, 2016). For *Schistosoma* larvae, it has been suggested that their fucosylated glycolipids are recognized by DCs in the skin (Figliuolo da Paz et al., 2019). These DCs react to the parasite with pro-inflammatory responses, yet the DC-SIGN activation induces an increase of anti-inflammatory cytokine IL-10. This increase in IL-10 prevents the generation of full adaptive responses which allows successful reinfection of the host (Mountford and Trottein, 2004). Similar responses have been observed in moDC for the DC-SIGN binding schistosomula EVs that contain ample fucosylated glycolipids (Kuipers et al., 2020). Glycolipids are insensitive to PNGase F treatment and we indeed still observed schistosomula EV-DC-SIGN interaction with the CLR-expressing cell lines after enzymatically removing the N-glycans (Figure 5D), similar to what we previously found for moDCs (Kuipers et al., 2020).

Although mouse MGL1 can recognize Le^X^ motifs, the human MGL cannot (van Vliet et al., 2008), and the observed interaction of schistosomula EVs with the human MGL in our experiments might be through the interaction of terminal GalNAc residues of the LDN-F epitopes present in schistosomula EV glycans (Table 1) (van Vliet et al., 2008) (van Vliet et al., 2008). The binding capacity of schistosomula EVs to MGL is limited, in contrast to the interaction of the adult worm EVs with this receptor (Figure 5). This finding is corroborated by the high abundance in adult worm EV of LDN motifs and the Tn antigen (Figure 5E), which are the two major ligands of the MGL (van Kooyk et al., 2015). The MGL is mainly associated with tolerogenic DCs and macrophages and MGL stimulation by the Tn antigen strongly increases IL-10 release (van Vliet et al., 2013). MGL is superior to DC-SIGN in the ability to generate IL-10 producing regulatory T cells after DCs stimulation (Hirata et al., 2016) and is mainly investigated for its role in tumor immunology (Valverde et al., 2020). Tumor cells are known to have altered glycosylation and the presence of the Tn antigen has been correlated to poor clinical outcome (Brockhausen, 2006). Although it has been shown that helminths such as *S. mansoni* stimulate the generation of regulatory cells (Maizels and McSorley, 2016), an exact role for the MGL during helminth infection has not been elucidated so far (van Kooyk et al., 2015). We therefore hypothesize that the adult worms release EVs to stimulate immune tolerance *via* interaction with the MGL. For example, human monocytes increase their MGL expression in the presence of IL-4 (Raes et al., 2005), a Th2 cytokine that accumulates in the granuloma induced by eggs trapped in the liver, which in turn links to the increased presence of alternatively activated macrophages (M2). It is therefore possible that adult worm EVs contribute to protecting the host from egg-derived toxins *via* MGL-induced tolerance (Costain et al., 2018). Interestingly, also schistosome egg-derived glycoprotein kappa-5 contains the LDN motif, as well as LDN-F. However, in addition to MGL, kappa-5 has been shown to interact with DC-SIGN and MR on the basis of the fucosylated LDN variant (Meevissen et al., 2012). Another observation for possible adult worm EV-induced tolerance comes from vaccination studies. Whereas recombinant *Schistosoma* TSP2 is a potential vaccine candidate against schistosomiasis (Mekonnen et al., 2020), initial vaccine studies with complete adult worm EVs showed no clear protection thus far (Kifle, 2020). This low efficacy of TSP2-containing worm EVs might be due to tolerogenic properties induced by EV glycosylation.

There are profound differences between the *Schistosoma* life stages. The schistosomulum has to find a way *via* the skin to the lungs and fight off the first line of defense of the host immune system while establishing infection and maturing into adult worms. Once developed into adult worms, the parasites reside in the veins near the liver and the gut where they need to establish a chronic infection and stay alive for years if untreated. Hence, the adult worms utilize different strategies than the larvae to evade or limit host responses. These differences are not only reflected in the morphology and glycosylation of the adult worms and schistosomula themselves, but also their released EVs. Furthermore, we found that glycosylation patterns differ between EVs and EV-depleted E/S (Table 1). This suggests that EVs from these parasites have a distinct contribution to infection and survival within their host. Studying the interactions of schistosome EVs with host CLRs not only increases our understanding of the roles of CLRs during helminth infections, it can also tell us how we could target these CLRs to either generate immune activation to fight infections and tumors or to induce immunotolerance to dampen unwanted inflammations.

## Data Availability

The raw data supporting the conclusion of this article will be made available by the authors, without undue reservation.
