# Supplementary material for: Life stage-specific glycosylation of extracellular vesicles from Schistosoma mansoni schistosomula and adult worms drives differential interaction with C-type lectin receptors DC-SIGN and MGL
Source: Front Mol Biosci. 2023 Mar 15;10:1125438. doi: 10.3389/fmolb.2023.1125438 (PMC10050886; doi:10.3389/fmolb.2023.1125438)
Supplement: Supplementary file 1 [file DataSheet1.pdf]

Supplementary Material

Life stage-specific glycosylation of extracellular vesicles from *Schistosoma mansoni* schistosomula and adult worms drives differential interaction with C-type lectin receptors DC-SIGN and MGL

Marije E. Kuipers, D. Linh Nguyen, Angela van Diepen, Lynn Mes, Erik Bos, Roman I. Koning, Esther N.M. Nolte-'t Hoen, Hermelijn H. Smits, Cornelis H. Hokke*

*** Correspondence:** Prof. C. H. Hokke, C.H.Hokke@lumc.nl

1 Supplementary Figures and Tables

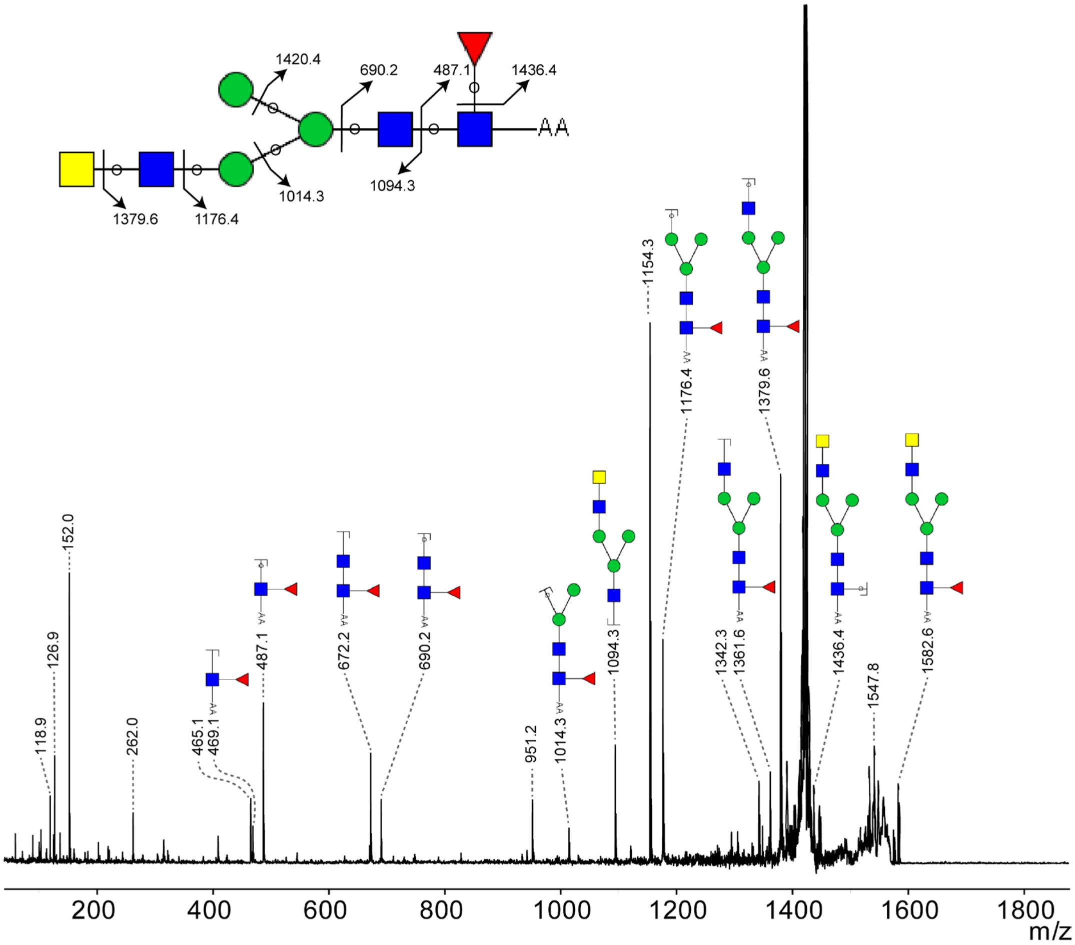

Supplementary Figure S1. MS/MS fragmentation of 1582 *m/z* to confirm LDN antenna
MS/MS fragmentation of 1582 *m/z* by MALDI-TOF/TOF of PNGase F released and AA-labeled N-glycans of adult worm EVs to confirm the presence of a single LDN antenna. Peaks are labelled with their monoisotopic masses. Green circle, mannose; yellow circle, galactose; blue square, N-acetylglucosamine; yellow square, N-acetylgalactosamine; red triangle, fucose.

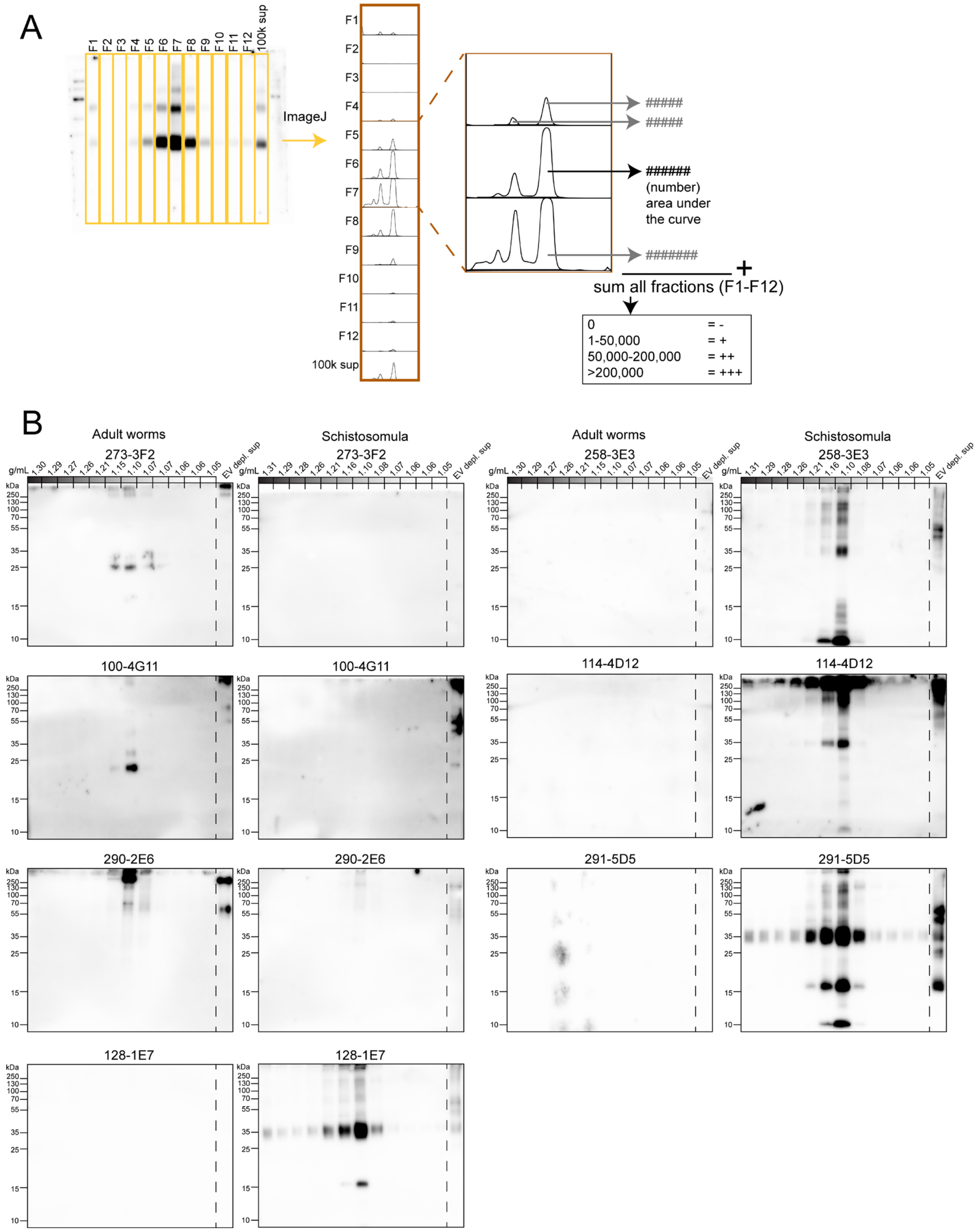

Supplementary Figure S2. Western blots of EV-associated glycoconjugates and their analysis by ImageJ.

Analysis workflow of measured blots in ImageJ (A). Area under the curve of the 12 gradient fractions were added together to form one number, EV-depleted (100k sup) supernatant had its own number. Full western blots of each antibody and life stage (B). Blots are representative for 2-3 biological replicates. 273-3F2 detects LDN; 100-4G11 detects tri-mannosyl core; 290-2E6 detects LDN-F; 128-1E7 detects F-GlcNAc, F-LDN, and F-LDN-F; 258-3E3 detects F-GlcNAc; 114-4D12 detects DF-GlcNAc and TF-GlcNAc; 291-5D5 detects F-LDN and F-LDN-F. Full description of these glycans and their linkage can be found in the legend of table 1.

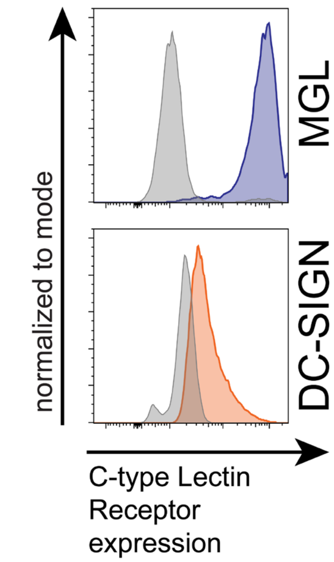

Supplementary Figure S3. MGL and DC-SIGN receptor expression on CLR expressing cell lines. CHO (MGL) and K562 (DC-SIGN) without the CLR vector are shown in grey.
